# Open Drug Discovery Toolkit (ODDT): a new open-source player in the drug discovery field

**DOI:** 10.1186/s13321-015-0078-2

**Published:** 2015-06-22

**Authors:** Maciej Wójcikowski, Piotr Zielenkiewicz, Pawel Siedlecki

**Affiliations:** Institute of Biochemistry and Biophysics PAS, Pawinskiego 5a, 02-106 Warsaw, Poland; Department of Systems Biology, Institute of Experimental Plant Biology and Biotechnology, University of Warsaw, Miecznikowa 1, 02-096 Warsaw, Poland

**Keywords:** Virtual screening, Statistical methods, Receptor-ligand interactions, Toolkit, Programming, Machine learning, Scoring function

## Abstract

**Background:**

There has been huge progress in the open cheminformatics field in both methods and software development. Unfortunately, there has been little effort to unite those methods and software into one package. We here describe the Open Drug Discovery Toolkit (ODDT), which aims to fulfill the need for comprehensive and open source drug discovery software.

**Results:**

The Open Drug Discovery Toolkit was developed as a free and open source tool for both computer aided drug discovery (CADD) developers and researchers. ODDT reimplements many state-of-the-art methods, such as machine learning scoring functions (RF-Score and NNScore) and wraps other external software to ease the process of developing CADD pipelines. ODDT is an out-of-the-box solution designed to be easily customizable and extensible. Therefore, users are strongly encouraged to extend it and develop new methods. We here present three use cases for ODDT in common tasks in computer-aided drug discovery.

**Conclusion:**

Open Drug Discovery Toolkit is released on a permissive 3-clause BSD license for both academic and industrial use. ODDT’s source code, additional examples and documentation are available on GitHub (https://github.com/oddt/oddt).

**Electronic supplementary material:**

The online version of this article (doi:10.1186/s13321-015-0078-2) contains supplementary material, which is available to authorized users.

## Background

Over the past decades, in silico drug discovery has become an important element augmenting classical medicinal chemistry and high throughput screening. Many novel computational chemistry methods were developed to aid researchers in discovering promising drug candidates. In recent years, much progress has been made in areas such as scoring functions, similarity search methods and statistical approaches (for review see [1, 2]). By contrast to computational chemistry, cheminformatics remains a relatively young field that suffers from many “early age diseases”, such as lack of standardization, particularly regarding data interchangeability and manipulation and reproducibility of results. To complicate the situation even more, format implementations usually have some additional, non-standard, software-oriented extensions (PDBQT is one prime example). Hardcoding a format into scientific software is also more common than using higher level toolkits, such as OpenBabel [3], RDKit [4], and OpenEye [5].

Some of the most popular and successful methods in drug discovery are structure-based. Structure-based methods are commonly employed to screen large small-molecule datasets, such as online databanks or smaller sets such as tailored combinatorial chemistry libraries. These techniques, from molecular docking to molecular mechanics to ensemble docking, employ scoring processes that are crucial for decision making. Empirical scoring functions use explicit equations based on physical properties of available ligand-receptor complexes. Knowledge-based scoring functions may additionally or exclusively use other types of interaction quantities that are parameterized using training set(s) to fit the data (for review see: [6, 7]). Currently, much effort is directed towards machine learning, which is most helpful in elucidating non-linear and non-trivial correlations in data. NNScore [8], Rfscore [9], and SFCscore [10] are among the most distinguished examples. However there are only a few freely accessible scoring functions and even fewer that are fully open source.

Analyzing output data, particularly when working with large scale virtual screening, can be a tedious and labor-demanding task that incorporates human error. Commercial software facilitate output data analysis to some extent, but there are also open source/free software solutions, such as VSDMIP [11] or DiSCuS [12], which are particularly designed for processing “big data”. However, the field is still missing a coherent, open source solution that will guide the researcher in building a custom cheminformatics pipeline, tailored for specific project needs. Therefore, we sought to develop a comprehensive open source small-molecule discovery platform for both researchers designing their own pipelines or developing new drugs. To achieve this goal, we have reviewed state-of-the-art tools and algorithms and united them in one coherent toolkit. When the use of open-source tools was not possible, the algorithms were reimplemented using open source software. This approach will make the in silico discovery process more scalable, cost-effective and easier to customize. We believe, that making software open is especially important to ensure data reproducibility and to minimize technology costs. Open-source software model allows numerous individuals to contribute and collaborate, on creating opportunities for novel tools and algorithms to be developed.

## Implementation

The Open Drug Discovery Toolkit (ODDT) is provided as a Python library to the cheminformatics community. We have implemented many procedures for common and more sophisticated tasks, and below we review in more detail the most prominent. We would also like to emphasize that by making the code freely available through a BSD license, we encourage other researchers and software developers to implement more modules, functions and support of their own software.

### Molecule formats

Open Drug Discovery Toolkit is designed to support as many formats as possible by extending the use of Cinfony [13]. This common API unites different molecular toolkits, such as RDKit and OpenBabel, and makes interacting with them more Python-like. All atom information collected from underlying toolkits are stored as Numpy [14] arrays, which provide both speed and flexibility.

### Interactions

The toolkit implements the most popular protein-ligand interactions. Directional interactions, such as hydrogen bonds and salt bridges, have additional strict or crude terms that indicate whether the angle parameters are within cutoffs (strict) or only certain distance criteria are met (crude). The complete list of interactions implemented in ODDT consists of hydrogen bonds, salt bridges, hydrophobic contacts, halogen bonds, pi-stacking (face-to-face and edge-to-face), pi-cation, pi-metal and metal coordination. These interactions are detected using in-house functions and procedures utilizing Numpy vectorization for increased performance. Calculated interactions can be used as further (re)scoring terms. Molecular features (e.g., H-acceptors and aromatic rings) are stored as a uniform structure, which enables easy development of custom binding queries.

### Filtering

Filtering small molecules by properties is implemented in ODDT. Users can use predefined filters such as RO5 [15], RO3 [16] and PAINS [17]. It is also possible to apply project-specific criteria for MW, LOGP and other parameters listed in the toolkit documentation. See Example 1 in the “Results and discussion” section for more details on how to use filtering.

### Docking

Merging free/open source docking programs into a pipeline can be a frustrating experience for many reasons. Some programs, like Autodock [18] and Autodock Vina [19], do not support multiple ligand inputs, where some other programs output scores to separate files (e.g., GOLD [20]) or even directly print to the console. Additional effort is required for re-scoring output ligand-receptor conformations in other software. Every in-silico discovery project is flooded with custom procedures and scripts to share data between programs. The docking stack within ODDT provides an easier path with the use of a common docking API. This API allows retrieving output conformations and their scores from various widely-used docking programs. The docking stack also supports multi-threading virtual screening tasks independently of underlying software, helping to utilize all available computational resources.

### Scoring

Open Drug Discovery Toolkit provides a Python re-implementation of two machine learning-based functions: NNscore (version 2) and RFscore. The training sets from its original publication were used for the RFscore function [9]. For NNScore, neither the training set nor the training procedure was made available by authors, other than a brief description [8]. To bring support for NNScore, we used ffnet  [21]. The training procedure for NNscore was reimplemented in ODDT and should closely reproduce the resulting ensemble of neural networks. The training data are stored as csv files, which are used to train scoring functions locally. After the initial training procedure, the scoring function objects are stored in pickle files for improved performance.

Machine learning scoring functions consist of four main building blocks: descriptors, model, training set and test set. ODDT provides a workflow for training new models, with additional support for custom descriptors and custom training and test sets. Such a design allows not only the use of the toolkit to reproduce scores (or reimplement scoring functions) but also enables the researcher to develop their own custom scoring procedures. Finally, if random seeds are defined, the scoring function results in ODDT are fully reproducible.

The ability to assess the predictive performance of scoring function (or scoring procedures) is of utmost importance. ODDT provides various ways to accomplish these tasks. One approach may use the area under receiver operating characteristics curve (ROC AUC and semi-log ROC AUC) and the enrichment factor (EF) at a defined percentage. These methods can be applied for every scoring function (and their combination) when training/test sets or active/inactive sets are supplied. Two other methods to test scoring function(s) performance include internal k-folds and leave one out / leave *p* out (LOO/LPO) cross-validation, both of which are particularly useful to detect model overfitting. These methods are available in ODDT through the sklearn python package [22].

### Statistical methods

Modeling the relationship between chemical structural descriptors and compound activities provides insight into SAR. Ultimately, such models may predict screening outcomes of novel compounds, guiding future discovery steps. Because some screening data are linear by their nature, simple regressors can be applied to find correlations (e.g., comparative molecular field analysis, CoMFA [23]). We implemented two straightforward regressions which that are widely used in cheminformatics, both in ligand and structure-based methods: multiple linear regression and partial least squares regression.

Nonlinear, more complex data are better assessed by machine learning models. Two forms of machine learning models are particularly important in drug discovery: (1) regressors for continuous data, such as IC50 values or inhibition rates, and (2) classifiers applied to multiple bit-wise features or ligands *tagged* as active/inactive (e.g., NNScore 1.0). ODDT employs sklearn as the main machine learning backend because it has a mature API and good performance. In some cases when neural networks are required, ODDT mimics the sklearn API and instead uses ffnet [21]. The current version of our toolkit provides machine learning models that are widely used in cheminformatics and drug discovery: (1) random forests, (2) support vector machines, and (3) artificial neural networks (single and multilayer). These models have been shown to provide great guidance when assessing protein-ligand complexes in the development and application of various scoring functions [8–10] and in SAR and QSAR (e.g., [24, 25]).

## Results and discussions

In this section, we provide examples of ODDT usage with code snippets. Our aim is to illustrate how one can utilize the toolkit for (a) preparing data for an in silico screening procedure, (b) score and rescore protein-ligand complexes, and (c) assess data quality and performance of different computational approaches for elucidating statistical correlations.

### Example 1: filtering, docking and re-scoring workflow

**Figure 1:**
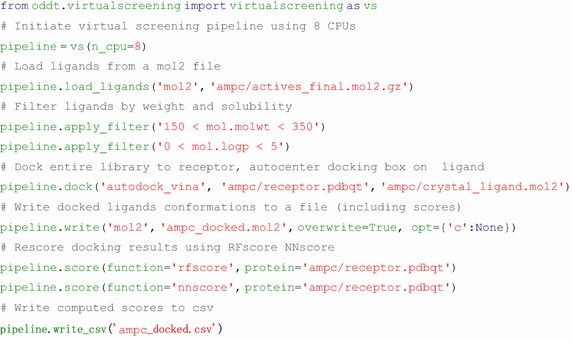
Code snippet illustrating ligand filtering, the docking procedure using the Autodock Vina engine, and rescoring with two machine learning functions: NNScore and RFscore.

In this code example, the researcher uses ODDT to dock a database of ligands with Autodock Vina and rescore the results with two independent scoring functions. First, he defines how many cores are available for this task (a 0 value will force all resources to be used). Next, a ligands library is loaded and two filtering steps are applied (for weight and solubility to be consistent with Lipinski’s “Rule of five” [15]. After filtering, the docking engine is specified (Autodock Vina) and its parameters can be defined (here default values are used, and the docking box is centered around a crystal ligand). In this example, the docked ligand conformations are written to a file for future examination. Two scoring functions are applied to the generated ligand-receptor conformations. The re-scoring results are finally written to a generic csv file for further analysis (Figure 1).

### Example 2: training and evaluating models for binding affinity datasets

In this example, the researcher is using a PDBbind dataset (ligand-receptor crystal structures along with experimentally-derived binding affinities (log Ki/Kd values) [26]. She wishes to train various prediction models on these data and then evaluate which model is the best predictor. (This workflow can also be used as a template to test and develop novel scoring functions and create custom, descriptor-based machine learning models).

In the first step, affinity values for both sets (training and test) are loaded from csv files. Then, all molecules and protein pockets (in sdf and pdb formats, accordingly) are read from the PDBbind 2007 directory (downloaded locally). Based on the csv files, these data are separated into training and test sets and close contact descriptors are generated (same as in RF-Score).

**Figure 2:**
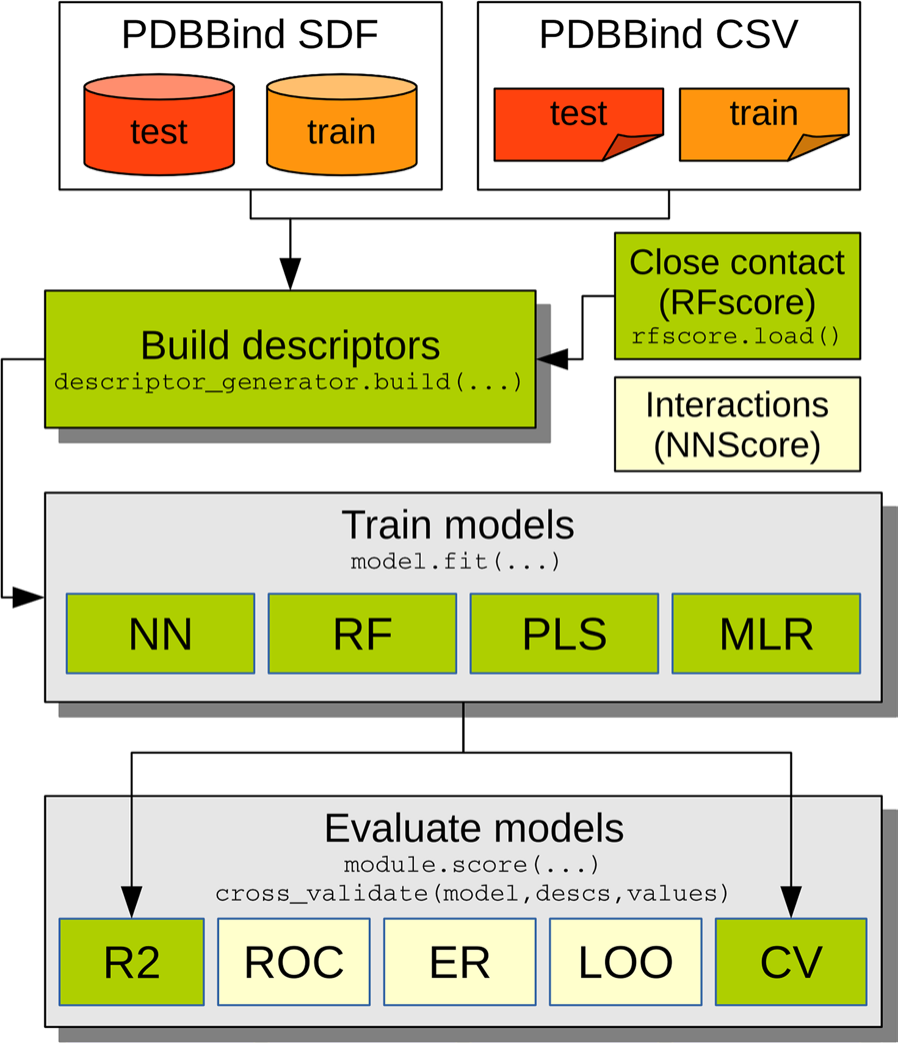
Workflow chart that illustrates how to select the best model for predicting compound activities based on the RF-Score descriptor. At each node there are methods/functions responsible for each calculation. Underlying code for this workflow is available in Additional file 1: (Snippet_2.ipnb).

**Figure 3:**
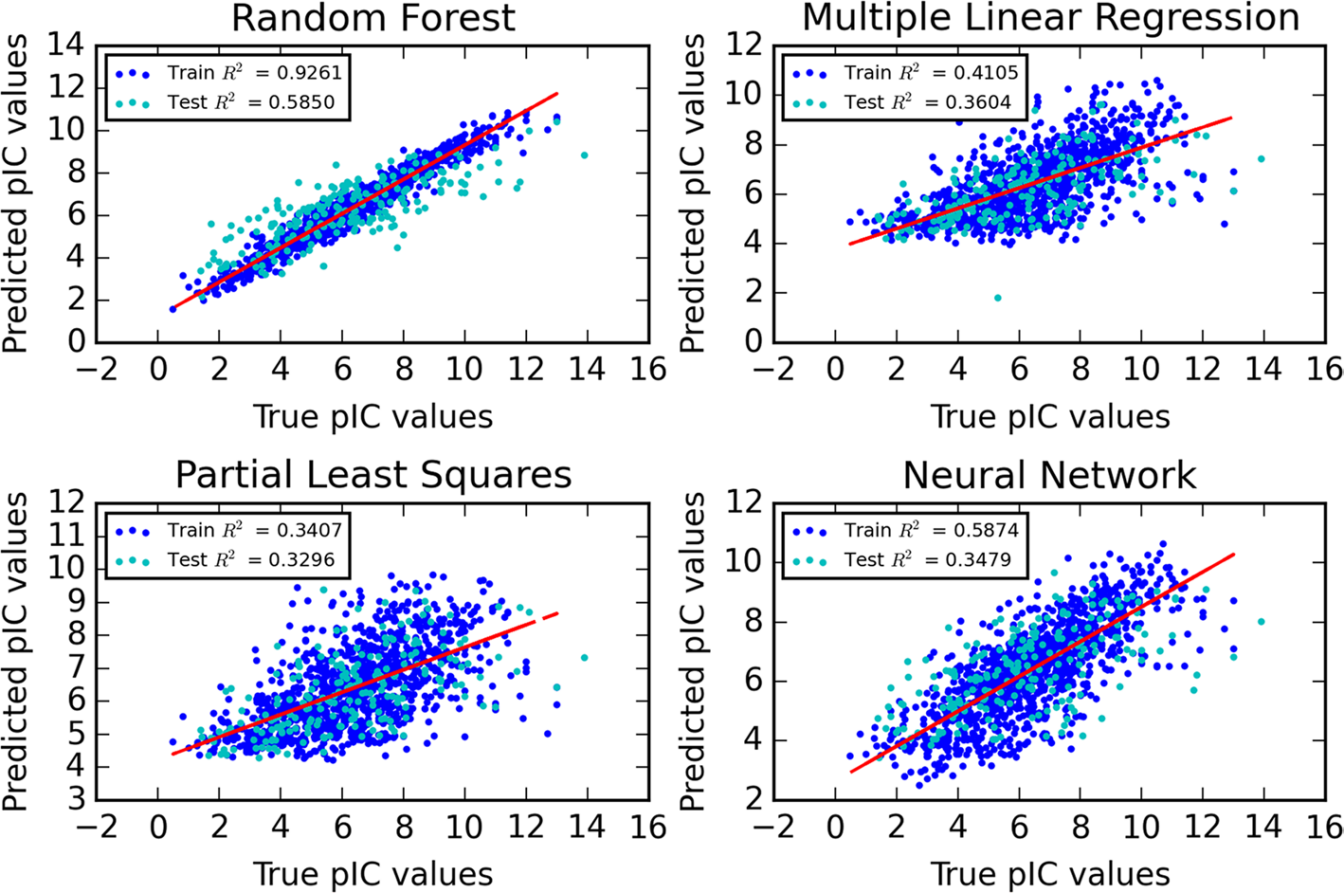
2D plots presenting the predicted and target affinities produced by specific models.

Then, the researcher trains various different regressor model types (random forests, support vector machines, neural networks and multiple linear regression). The performance of every scoring function is simultaneously estimated by computing the correlation coefficient (R) between the predicted and target affinities; additionally, a 2D plot is drawn. To check whether the models are overfit, 10-fold cross validation is performed on the joined test and training sets to derive the mean and standard deviation of 10 correlation coefficients of the cross validation sets (Figures 2, 3).

### Example 3: training classifiers to distinguish active from inactive compounds based on DUD-E

This code snippet illustrates how to determine which fingerprint descriptor is the most suitable for describing active compounds for the AMPC protein by using DUD-E’s subset of actives, inactives and decoys. The random forest classifier model is trained using various fingerprints implemented both in RDKit and OpenBabel.

Firstly, molecules for actives, inactives, decoys and marginal actives (treated as inactives for training) are read from SMILES files. Next, a wide range of fingerprints is built for all molecules: OpenBabel: fp1, fp2, MACCS; RDKit: rdkit (default), morgan, layered.

**Figure 4:**
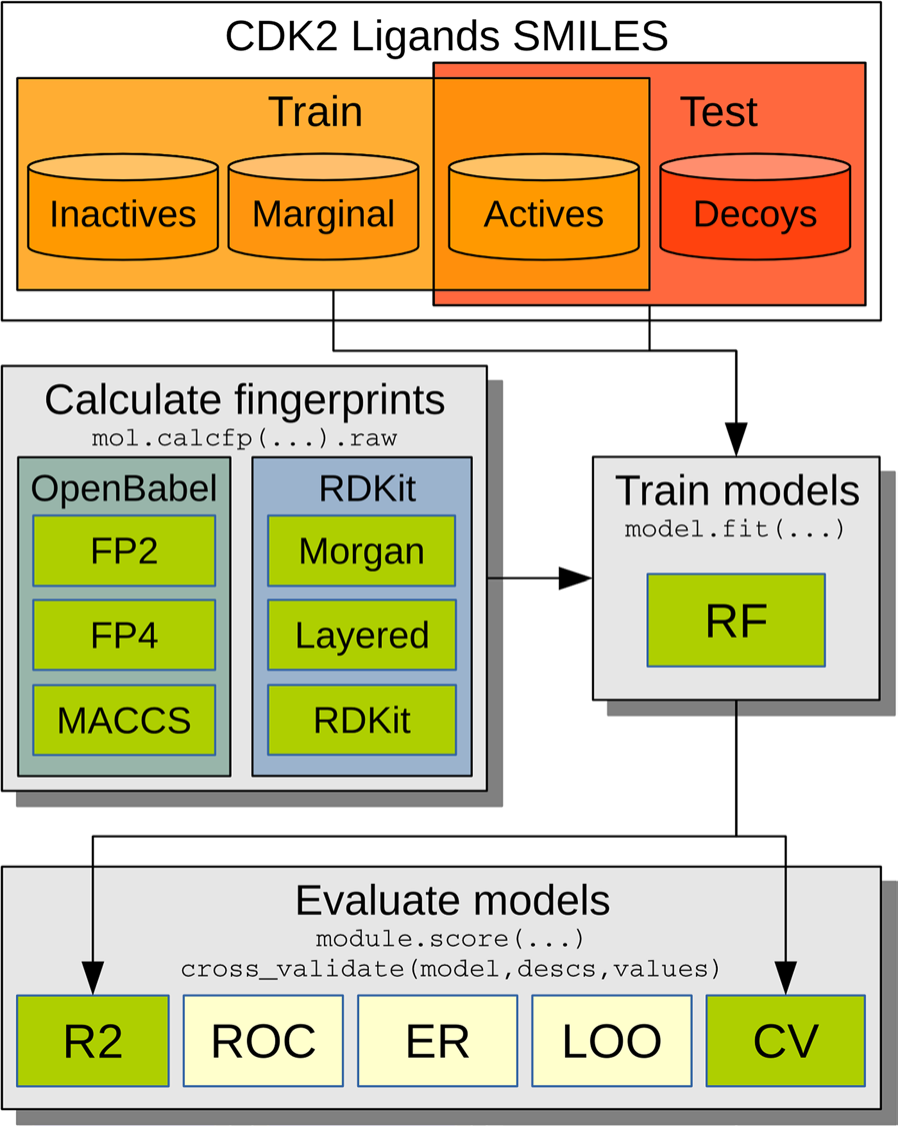
Workflow to assess the performance of using specific fingerprints for distinguishing actives from a library of substances. At each node there are methods/functions responsible for each calculation. The code for this workflow is available in Additional file 1: (Snippet_3.ipnb).

Secondly, a random forest classifier model is fit on all computed fingerprints, and the quality of the trained model is assessed by a correlation coefficient (R). Additionally, trained models are cross-validated to examine overfitting. From such a short analysis, one can conclude that in the presented case, morgan fingerprints yields the best results (R^2^ = 0.99) in classifying active molecules in the benchmarking sets taken from DUD-E (Figure 4).

## Conclusion

In this article, we introduce an out-of-the-box solution for building in-silico screening and data elucidation pipelines. The solution is flexible and provides a selection of useful tools, some of which are implemented for the first time. The three workflows illustrated in this paper demonstrate how one can use the toolkit to quickly prepare, filter, and screen data and apply various statistical methods to elucidate relationships.

## Availability and requirements

ODDT (Open Drug Discovery Toolkit) is available at https://github.com/oddt/oddt

Operating system(s): platform independent

Programming language: Python

Other requirements:

- at least one of the toolkits:
  - OpenBabel (2.3.2+),
  - RDKit (2012.03)
- Python (2.7+)
- Numpy (1.6.2+)
- Scipy (0.10+)
- Sklearn (0.11+)
- ffnet (0.7.1+), only for neural network functionality.

License: 3-clause BSD,

Any restrictions to use by non-academics: none.

## Additional file

Additional file 1: Snippets.zip—IPython notebooks containing the code for each example.

## Abbreviations

CADD: computer aided drug discovery
ODDT: Open Drug Discovery Toolkit
EF: enrichment factor
ROC: receiver operating characteristic
AUC: area under curve
LOO: leave one out
LPO: leave p out
USR: ultra-fast shape recognition
SAR: structure-activity relationship
